# The association between system-justifying ideologies and attitudes toward the social market economy in Germany

**DOI:** 10.1007/s12144-023-04483-7

**Published:** 2023-03-31

**Authors:** Alexander Jedinger, Simone Kaminski

**Affiliations:** 1 GESIS – Leibniz Institute for the Social Sciences, Cologne, Germany; 2 Munich University of Applied Sciences, Munich, Germany

**Keywords:** Political ideology, Social market economy, Capitalism, Right-wing authoritarianism, Social dominance orientation, System justification

## Abstract

Although the legitimacy of an economic system is often dependent on citizen support, psychological research has paid little attention to attitudes toward economic systems. In the present study, we examined the link between two system-justifying ideologies, namely, right-wing authoritarianism (RWA) and social dominance orientation (SDO), and attitudes toward the social market economy in Germany. Drawing on system justification theory, we hypothesized that RWA would be positively and SDO negatively associated with support for the social market economy because the social component of the German economic system conflicts with beliefs inherent in SDO favoring a group-based hierarchy. Based on a quota sample of German adults (*N* = 886), we found support for the predicted associations of both system-justifying ideologies with economic system support, except that RWA was negatively associated with support for the welfare component of the social market economy. However, the positive relationship of RWA with support for the social market economy only emerged after SDO was statistically controlled, suggesting a suppressor situation. These findings demonstrate that system-justifying ideologies bear different relations to pro-market attitudes depending on the type of economic regime. Implications for system justification theory are discussed.

**Supplementary Information:**

The online version contains supplementary material available at 10.1007/s12144-023-04483-7

## Introduction

Given the economic turmoil of recent decades, the question of mass support for the free-market economy has once again attracted scholarly attention (e.g., Azevedo et al., 2019; Bettache & Chiu, 2019; Cichocka & Jost, 2014). A popular explanation for the acceptance of the market economy is based on the notion that people support free-market principles to the extent that they serve their material self- and group-interests. In fact, some studies have indicated that economic deprivation and lower socioeconomic status are associated with hostility toward market competition (Hayo, 2004; Kotzian, 2015; Landier et al., 2008; Tufiș, 2010). However, past research suggests that political ideology and religiosity are often more powerful predictors of economic system support than subjective or objective indicators of material interests such as income or occupational status. For instance, conservatism and Protestant religious faith are positively associated with support for laissez-faire capitalism (Barker & Carman, 2000; Deckman et al., 2017; Hayward & Kemmelmeier, 2011). These findings align with recent studies that link pro-market attitudes with the endorsement of system-justifying ideologies such as authoritarianism and social dominance orientation (Azevedo et al., 2019; Beattie et al., 2019).

In the current study, we extend past research by examining the associations of authoritarianism and social dominance orientation with attitudes toward the social market economy in Germany. Recent research suggests that the relationship between system-justifying ideologies and politico-economic attitudes is contingent on country-level differences in social and economic institutions (Brandt & Reyna, 2017). Germany is an informative case because the market economic system is characterized by a strongly developed welfare system to shield people against excessive market-based risks (Müller-Armack, 1965; Muresan, 2014). Drawing on system justification theory (Jost, 2020), we test the hypothesis that authoritarianism is positively related to pro-market attitudes among the German population. However, in light of the aversion of high social dominators to redistributive principles, we expect that social dominance orientation will be negatively associated with support for the social component of the German economic system.

## System justification theory

In 1994, Jost and Banaji (1994) proposed a psychological theory of system justification as an advancement or complement to social identity theory (Tajfel & Turner, 1986) and social dominance theory (Sidanius, 1993). System justification theory (SJT) is often raised to explain why people defend the status quo and bolster the social, political, and economic systems in which they live and work even though these arrangements contradict their material interests or bring more disadvantages than advantages for them (Osborne et al., 2019).

According to this approach, people have a general psychological tendency to perceive the systems to which they belong as fair and legitimate because, in this way, central psychological needs to reduce uncertainty and threat are satisfied (Jost, 2020; Jost et al., 2004; Jost & van der Toorn, 2012). This leads them to adapt system-justifying ideologies to legitimize material differences between social status groups and reinforce the economic status quo. These system-justifying beliefs include the Protestant work ethic (Jost & Hunyady, 2002), meritocractic beliefs (Jost et al., 2003; McCoy & Major, 2007), fair market ideology (Jost et al., 2003), and economic system justification (Jost & Thompson, 2000). In turn, people who embrace system-justifying ideologies are more likely to support conservative economic policies and oppose welfare policies that level perceived status inequalities (Jost et al., 2017; Wakslak, Jost, Tyler, & Chen, 2007). Moreover, system justification positively affects individual self-esteem and life satisfaction, at least in the short term (Goudarzi et al., 2020; Harding & Sibley, 2013; McCoy et al., 2013). System justification also reinforces the negative stereotyping of disadvantaged groups. For example, economically disadvantaged groups are attributed characteristics such as laziness and lack of intelligence to rationalize their lower social status in society (Jost & Banaji, 1994; Kay et al., 2007).

The extent to which people adhere to system-justifying beliefs depends on various dispositional and situational factors. At the personal level, an epistemic need for certainty, existential needs to manage threats, and the desire for positive social relationships promote system justification tendencies. Consistent with this, empirical research shows that individuals with a high need for cognition, low openness to new experiences, and strong mortality salience tend to perceive their surrounding economic system as fair and just (Hennes et al., 2012). Furthermore, the extent of system justification is also influenced by situational factors, especially when the prevailing economic arrangement is threatened or criticized (Banfield et al., 2011; Becker & Sparks, 2016; Ledgerwood et al., 2011; Liviatan & Jost, 2014).

## System-justifying ideologies and support for free markets

The present investigation focuses on two related but distinct system-justifying ideologies, right-wing authoritarianism (RWA) and social dominance orientation (SDO). Over the years, a consensus emerged that political ideologies are not characterized by a one-dimensional left-right scheme but consist of two basic dimensions pertaining to conformity, security, and traditionalism on the one hand and dominance, hierarchy, and group-based inequality on the other hand (Claessens et al., 2020; Jost, 2021). Although multiple variables representing individual difference have been proposed to reflect the dual dimensions of ideology, RWA and SDO are the most robust belief systems that predict a wide range of attitudinal outcomes, including social and economic policy preferences (Duckitt, 2001; Duckitt & Sibley, 2009). RWA encompasses obedience to established authorities, conformity to legitimate norms within a society, and aggressiveness toward individuals who deviate from these norms (Altemeyer, 1996). SDO describes the belief that the relationships between social groups should be hierarchically organized and that some groups are superior to others (Pratto et al., 1994). While authoritarianism is primarily concerned with reinforcing cohesion, order, and control within social groups, social dominance addresses the distribution of power and resources between social groups (Duckitt & Sibley, 2009).

System justification theory proposes that RWA and SDO serve as system-justifying beliefs among more domain-specific ideologies such as economic system justification (Jost & Hunyady, 2005). Economic system justification, typically measured with the Economic System Justification (ESJ) scale, is defined as “the general ideological tendency to legitimize economic inequality” (Jost & Thompson, 2000, p. 225) and thereby echoes the underlying principles of the free-market economy. However, we consider RWA and SDO factors causally prior to economic system justification and related constructs because they are more abstract and rooted in basic personality traits (for a similar argument, see Vargas-Salfate et al., 2018).

Authoritarians support the status quo, which is considered legitimate insofar as recognized authorities sanction it in society. In this respect, they support the current economic system, regardless of its concrete institutional design, because they reject social change. Consistent with this, studies suggest that the relationship between authoritarianism and economic attitudes is contingent on the socioeconomic system. In Western capitalist societies, authoritarian predispositions are associated with right-wing economic attitudes (Bobbio et al., 2010; Cornelis & Van Hiel, 2006), whereas in former communist societies, they are linked to left-wing economic positions (Duriez et al., 2005; Kossowska & Hiel, 2003; McFarland et al., 1996; Radkiewicz, 2017; Van Hiel & Kossowska, 2007). In contrast, people scoring high in social dominance orientation appear to have a natural affinity for free-market arrangements because capitalist economies inherently foster intergroup competition and legitimize inequality by meritocratic ideals. Thus, studies have demonstrated that SDO consistently fuels support for economic conservatism, capitalism, and opposition to redistribution (Bobbio et al., 2010; Cornelis & Van Hiel, 2006; Jost & Thompson, 2000; Radkiewicz, 2017; Van Hiel & Kossowska, 2007). Moreover, several recent studies show that both RWA and SDO are unique predictors of economic system justification, neoliberal beliefs, and support for laissez-faire capitalism (Azevedo et al., 2019; Bay-Cheng et al., 2015; Beattie et al., 2019; Jost, 2019; Jost et al., 2003).

## The case of Germany

The German economic system called “Soziale Marktwirtschaft” (*social market economy*) was implemented in 1948 by Ludwig Erhard, former Minister for Economic Affairs (1949–1963) and later Chancellor of the Federal Republic of Germany (1963–1966) (Muresan, 2014). According to Müller-Armack (1989, p. 83), the concept of a social market economy “aims to combine, on the basis of a competitive economy, free initiative and social progress.” This means that the state replenishes the system of the pure market economy, which is characterized by active competition in the market, with social elements such as compulsory health insurance or compulsory unemployment insurance to protect the economically vulnerable.

The implementation of the social market economy in Germany after the Second World War was closely related to the goal to achieve secured prosperity for the entire population but on the condition of competition. In the following years, the success of this system appeared in the quick economic recovery of West Germany, the so-called economic miracle, or Miracle on the Rhine. The model of the social market economy in Germany combines economic freedom (i.e., enterprise opportunity, private property and free competition) with social protection that strives for social balance and aims for collective security, social redistribution of income, solidarity, and subsidiarity (i.e., the state intervenes only if an individual’s possibilities are insufficient) (Muresan, 2014).

Public support for the social market economy is high. According to findings by the German opinion research Institute IfD Allensbach in 2021, 54% of the respondents of a quota-representative sample reported that they have a “good opinion” about the social market economy, despite the COVID-19 crisis and the associated economic consequences (Institut für Demoskopie Allensbach, 2021). A representative survey on behalf of the Association of German Banks (2021) reached a similar result: 78% of the respondents consider that the social market economy has proven itself. However, these results are based on one-item, nominal scaled measures, and until now, relationships with ideological orientations such as RWA and SDO were not examined.

## Overview and hypotheses

The present study aims to explore the association of RWA and SDO with attitudes toward the social market economy in Germany. We extend previous studies on the psychological underpinnings of economic system support in several ways. First, most studies considering the association between RWA, SDO, and the endorsement for the existing economic system originate in the United States. That is, they focus on liberal market economies with little or no governmental control. In contrast, the relation of system-justifying ideologies with support for social market economies characterized by more substantial interventions into the free market remains unclear. Second, most studies predominantly rely on the ESJ scale. However, the ESJ scale measures psychological defense on behalf of the system but not individuals’ attitudes toward the economic system as such. Becker and Sparks (2016) demonstrated that the scale is not one-dimensional but instead captures the perceived fairness of the system, opposition to redistribution of resources, and the belief that inequality is natural. Thus, the ESJ scale mixes explanatory factors such as meritocratic beliefs or attributions of poverty with the measurement of actual system support and thereby obscures relationships with ideological orientations. We address this limitation by administering a new measure of attitudes toward the social market economy that allows us to examine system support directly (see below). We hypothesize that RWA will be positively associated with support for the social market economy (Hypothesis 1). In contrast to the studies mentioned above, we hypothesize that SDO will be negatively associated with support for the social market economy (Hypothesis 2) because the social component of the German economic system distinguished by a strongly developed welfare system conflicts with preferences for group-based hierarchy and inequality.

## Method

### Participants and procedure

We conducted a cross-sectional web survey of German adults (*N* = 1,114) from June 4 to 10, 2021, recruited through the Mingle Research Panel (www.mingle.respondi.com). Participants were selected to reflect the German population over 18 years in terms of age, gender, education, and region. After giving their informed consent, participants were assessed on measures of economic system support, system-justifying ideologies, economic deprivation, social status, demographics, and other measures unrelated to the present investigation. We excluded participants who failed to provide complete answers to the main study variables, which left us with a final sample of 886 participants (53.2% male, 35.4% university entrance level education) ranging in age from 18 to 85 years (*M* = 49.2, *SD* = 16.6).^1^ The median income category was 2,000–2,999 Euros. A comparison of the sample to the general German population and additional descriptive statistics for key variables can be found in the Supplementary Online Material.

**Table 1:** Brief Social Market Economy Attitude Inventory and Factor Loadings

Item		Factor Loadings		
		1	2	3
1.	The social market economy has proven its worth in Germany so far.	**0.75**	0.08	− 0.01
2.	The positive aspects outweigh the negative aspects of the social market economy.	**0.70**	0.14	0.03
3.	I support the principles of the social market economy.	**0.72**	0.06	− 0.00
4.	I feel like a winner of the social market economy.	0.08	**0.69**	0.01
5.	I benefit from the results of the social market economy.	0.16	**0.66**	0.03
6.	The social market economy results in the welfare state being exploited by many people.*	− 0.10	0.09	**0.51**
7.	The social component of the social market economy hinders the future viability of our country.*	0.19	− 0.12	**0.49**
	**Factor Correlations**			
	Factor 1: Diffuse Support	—		
	Factor 2: Perceived Personal Advantages	0.71	—	
	Factor 3: Welfare Component	0.25	0.08	**—**

*Note.* Pattern matrix using principal axis factoring and oblimin rotation with Kaiser normalization. Boldface indicates the highest factor loadings. Items marked with an asterisk are reverse coded. *N =* 790.

### Measures

#### Attitudes toward the social market economy

We used a brief version of the Attitude toward the Social Market Economy (ASME) scale. The original scale contained 25 items and was developed to assess different facets of opinions toward the German economic system, e.g., diffuse support for the social market economy and perceived personal advantages (Kaminski, 2008). In contrast to other instruments measuring attitudes toward free-market systems (e.g., McClosky & Zaller, 1984), the ASME scale includes support for the social component of the German market economy. The brief version contains seven items. Example items include statements such as, “The social market economy has proven its worth in Germany”, “I see myself as a winner of the social market economy” or “The social market economy leads to the welfare state being exploited by many people.” Participants responded to these items on a five-point scale (1 = *strongly disagree*, 5 = *strongly agree*). Because this measure is relatively new, we performed a principal axis factor analysis with oblique (oblimin) rotation of all seven items (see Table 2). The Kaiser–Meyer–Olkin coefficient indicates that the item selection is adequate for factor analysis (KMO = 0.81), and all interitem correlations are significantly different from zero, χ^2^(21) = 2107.21, *p* < .001. We extracted three factors as suggested by a parallel analysis (Horn, 1965) with 1,000 replications. The first factor reflects general (diffuse) support for the social market economy (three items, *M* = 3.62, *SD* = 0.88, Cronbach’s α = 0.85), the second factor indicates perceived personal advantages (two items, *M* = 3.03, *SD* = 0.95, Spearman-Brown coefficient = 0.79), and the third factor can be interpreted as support for the social component of the social market economy (two items, *M* = 2.84, *SD* = 0.93, Spearman-Brown coefficient = 0.52).

**Table 2:** Correlations among the Main Study Variables

	1	2	3	4	5	6	7	8	9
1. Diffuse Support	—								
2. Personal Advantages	0.63***	—							
3. Welfare Component	0.19***	0.11***	—						
4. Right-wing Authoritarianism	-0.01	0.05	-0.34***	—					
5. Social Dominance Orientation	-0.24***	-0.16***	-0.35***	0.39***	—				
6. Individual Deprivation	-0.31***	-0.40***	-0.08**	-0.03	0.06	—			
7. Collective Deprivation	-0.32***	-0.35***	-0.15***	-0.03	0.11**	0.44***	—		
8. Individual Relative Deprivation	-0.27***	-0.45***	-0.10**	-0.01	0.06	0.41***	0.34***	—	
9. Subjective Social Status	0.18***	0.28***	0.02	0.05	0.03	-0.45***	-0.21***	-0.38***	—
10. Household Income	0.16***	0.20***	0.03	-0.07*	-0.03	-0.37***	-0.10**	-0.25***	0.43***

*Note.* * *p* < .05, ** *p* < .01, *** *p* < .001; *N* = 886.

#### System-justifying ideologies

RWA (*M* = 3.19, *SD* = 0.70, Cronbach’s α = 0.80) was assessed using a nine-item scale (Beierlein et al., 2014). An example item is “We need strong leaders so that we can live safely in society.” Social dominance orientation (*M* = 2.14, *SD* = 0.83, Cronbach’s α = 0.72) was measured with a brief four-item version of the SDO scale (Stellmacher et al., 2005). An example item is “Some groups of people are simply inferior to other groups.” All items were rated on a five-point scale (1 = *strongly disagree*, 5 = *strongly agree*) and averaged to produce a composite score.

#### Economic deprivation

To capture levels of economic deprivation, participants completed three items adapted from the German General Social Survey (GESIS, 2021). Individual absolute deprivation was measured with the following item: “How would you evaluate your current economic situation?” (1 = *very good*, 5 = *very bad*; *M* = 2.81, *SD* = 0.92). Collective absolute deprivation was measured with the item “How would you evaluate the current general economic situation in Germany?” (1 = *very good*, 5 = *very bad*; *M* = 3.11, *SD* = 0.86). To gauge individual relative deprivation, participants were asked to rate on a five-point scale whether they receive their fair share compared to others (“Compared with how others live in Germany: Do you think you get your fair share, more than your fair share, much more, somewhat less or very much less than your fair share?”). Responses were given on a five-point scale (1 = *much more*, 5 = *much less*, *M* = 3.49, *SD* = 0.80).

#### Social Status

We used an adapted version of the McArthur Scale to assess participants’ subjective social status (Adler et al., 2000). Participants were asked to place themselves on a ladder that ranged from 1 to 10, where 10 represented people in Germany with the highest status and 1 represented people with the lowest status (*M* = 5.20, *SD* = 1.74). Objective social status was determined by monthly household income recoded to income quintiles (1 = *under 1.500 euro*, 2 = *1,500-1,999 euro*, 3 = *2,000–2,999* euro, 4 = *3,000–3,999 euro*, 5 = *4,000 euro or greater*, *M* = 2.86, *SD* = 1.40). Because many participants (8.9%) refused to report their household income, we imputed their income levels using predictive mean matching (Morris et al., 2014).

#### Covariates

We controlled for political ideology (1 = *extremely left*, 7 = *extremely right*; *M* = 3.94, *SD* = 1.07), political interest (1 = *not at all*, 5 = *very strong*; *M* = 3.29, *SD* = 1.07), age (*in years*), gender (1 = *male*, 0 = *female*), education (1 = *lowest secondary qualification, after nine years of schooling*, 2 = *intermediary secondary qualification, after ten years of schooling*, 3 = *higher secondary qualification, after 12 or 13 years of schooling*), and region of residence (1 = *East Germany*, 0 = *West Germany*).

## Results

Bivariate correlations for all variables are presented in Table 3. Most importantly, RWA was unrelated to diffuse support for the social market economy and perceived personal advantages within the system, which counters our theoretical expectations. In addition, RWA was unexpectedly related to lower levels of support for the welfare component of the social market economy. In line with our expectations, SDO was negatively related to all three dimensions of economic system support. Perceived material deprivation was also negatively associated with all dimensions of attitudes toward the social market economy, while subjective and objective indicators of social status were positively correlated with diffuse support and perceived personal advantages. The bivariate analysis thus provides preliminary evidence for Hypothesis 2 but no support for Hypothesis 1.

**Table 3:** Multiple Regression Predicting Attitudes toward the Social Market Economy

Predictor	Diffuse Support			Perceived Personal Advantages			Support for the Welfare Component	
	*B* (*SE*)	*Beta*		*B* (*SE*)	*Beta*		*B (SE)*	*Beta*
Right-wing Authoritarianism	0.11*	0.09		0.17***	0.13		-0.29***	-0.22
	(0.05)			(0.05)			(0.06)	
Social Dominance Orientation	-0.25***	-0.23		-0.16***	-0.14		-0.22***	-0.19
	(0.04)			(0.04)			(0.04)	
Individual Deprivation	-0.13***	-0.14		-0.18***	-0.18		-0.02	-0.02
	(0.04)			(0.04)			(0.04)	
Collective Deprivation	-0.14***	-0.14		-0.13**	-0.12		-0.08*	-0.08
	(0.04)			(0.04)			(0.04)	
Individual Relative Deprivation	-0.12**	-0.11		-0.33***	-0.28		-0.05	-0.04
	(0.04)			(0.04)			(0.04)	
Subjective Social Status	-0.01	-0.01		0.05	0.04		-0.02	-0.01
	(0.04)			(0.05)			(0.05)	
Household Income	0.03	0.04		0.00	0.01		-0.03	-0.04
	(0.02)			(0.02)			(0.02)	
Political Ideology	-0.03	-0.02		-0.10*	-0.07		-0.19***	-0.15
	(0.05)			(0.04)			(0.05)	
Political Interest	0.11***	0.13		0.04	0.04		0.04	0.04
	(0.03)			(0.03)			(0.03)	
Age	0.05	0.06		-0.01	-0.01		0.04	0.05
	(0.03)			(0.03)			(0.04)	
Male	0.04**	0.09		0.03	0.06		0.04**	0.09
	(0.01)			(0.01)			(0.02)	
Education (Ref.: Low Education)								
Medium Education	-0.04*	-0.09		-0.02	-0.05		0.01	0.01
	(0.02)			(0.02)			(0.02)	
High Education	0.01	0.03		0.02	0.05		0.04	0.08
	(0.02)			(0.02)			(0.02)	
East Germany	-0.04*	-0.08		-0.02	-0.03		-0.01	-0.01
	(0.02)			(0.02)			(0.02)	
Constant	0.79***			0.81***			0.80***	
	(0.06)			(0.05)			(0.06)	
Adj, *R*^2^	0.25			0.31			0.21	
* F*(14, 871)	22.38***			29.27***			16.47***	

*Note*: The entries are unstandardized OLS regression coefficients, robust standard errors in parentheses, and standardized coefficients. All continuous variables ranged from 0 to 1; *N* = 886.* *p* < .05, ** *p* < .01, *** *p* < .001

To examine the unique contribution of each predictor, we performed a series of multiple regression analyses with the three dimensions of attitudes toward the social market economy as dependent variables.^2^ We recoded all variables to range from 0 to 1 to facilitate interpretations of unstandardized predictors (Cohen et al., 1999).^3^ Regression diagnostics show no concerns for outliers or multicollinearity (all variance inflation factors were < 2.0). However, we employed Huber-White robust standard errors to correct for heteroskedasticity. The results of the regression analysis are reported in Table 1.

Across all models, we found evidence confirming that ideological predictors are closely linked with different facets of support for the social market economy. Consistent with the second hypothesis, SDO is strongly and negatively associated with all aspects of economic system support. People scoring higher on the SDO scale were less supportive of the overall concept of the SME, perceived the system as less beneficial to them, and strongly opposed the redistributive aspect of the SME. In contrast to the bivariate results, RWA emerged as a positive predictor of general system support and perceived personal advantages. People scoring higher on the RWA scale generally evaluate the social market economy system more positively and see greater personal benefits. Thus, statistically controlling for SDO revealed the expected positive association between RWA and support for the social market economy by removing shared variance between both ideological orientations. In other words, SDO is a suppressor that enhances the relationship between RWA and pro-market attitudes (see Paulhus et al., 2004). RWA purged of beliefs in group-based inequalities apparently became a system-supporting disposition.

Contrary to the first hypothesis, however, RWA was still negatively related to the welfare aspect of the social market economy, suggesting that authoritarian people are skeptical of the way the strongly developed welfare system is a complement to the free market. Although authoritarians and social dominators agree in their rejection of economic redistribution, highly authoritarians seem to separate more strongly between the idea of a market economy and its supplementation by social policy measures in the German model. Taken together, the multivariate results provide partial support for the first hypothesis.

Regarding the other predictors, we see that individual and collective economic deprivation is negatively associated with diffuse support and perceived personal advantages of the social market economy. Interestingly, feelings of economic discontent were unrelated to support for redistributing economic resources. Subjective social status and household income were not significantly associated with expressions of economic system support in the multivariate model. Among the controls, we observed that right-wing ideological identification was negatively related to perceived personal advantages and support for the welfare component of the SME. Political interest was only positively associated with diffuse support for the German economic system. The other control variables had no substantial relationships with economic system support.

### Discussion

The current study contributed to the literature by investigating the link between right-wing authoritarianism (RWA), social dominance orientation (SDO) and economic system support in a German context. Taking recourse to system justification theory, we proposed that RWA would be positively and SDO negatively associated with support for the social market economy because the welfare element of the German economic system conflicts with social dominators’ aversion to the leveling of social hierarchies. Our results lend support to the predicted relationships of both ideological orientations, with the exception that RWA was negatively associated with the endorsement of the welfare component of the social market economy. Contrary to our expectations, the positive association between RWA and two facets of attitudes toward the social market economy (diffuse support and perceived personal advantages) only arose after the shared variance of RWA with SDO was statistically controlled (but see footnote 1).

Our findings do not support system justification theory entirely. RWA and SDO are usually considered ideological orientations that foster pro-capitalist attitudes. However, most previous research was conducted in countries with a pronounced liberal market economy. These studies demonstrated a strong positive relationship between support for the free-market economy and system-justifying ideologies such as RWA and SDO (e.g., Azevedo et al., 2019; Beattie et al., 2019). Our results suggest that the nature of this relationship is strongly dependent on the economic and cultural context. The endorsement of the redistributive aspect of the German economic system runs counter to the tenets of SDO because participants scoring high in SDO prefer differences in social status between societal groups and favor group-based hierarchy (Pratto et al., 1994). Moreover, the social design of the German economic system seems to overlay all aspects of the social market economy because SDO was also negatively related to support for the overall concept of the social market economy and perceived personal advantages of the economic system.

Concerning RWA as a system-justifying ideology, we identified a suppressor situation in which adding SDO to a regression predicting economic system support enhances the predictive validity of RWA for diffuse support and the perceived personal advantages of the social market economy. Interestingly, after adding SDO to the regression equation, RWA was still a negative predictor of the welfare aspect of the social market economy. The observed suppressor effect is in line with recent research showing mutual suppression between social and economic conservatism that has often been associated with RWA and SDO (e.g., Costello & Lilienfeld, 2021). We suppose that people scoring high on the RWA scale are skeptical of the strongly developed welfare system as a complement to the free market. Including SDO suppresses the part of RWA that instigates skepticism toward the welfare state and reveals support for the competitive aspect of the German economic model. While RWA is generally conceived as a system-justifying belief system that supports the status quo, this does not seem to be true for redistributive policies, even if they are an integral part of the German market economic system. We speculate that this antipathy could have two reasons. First, authoritarians view social hierarchies as sources of stability and order (Jost, 2021). The redistribution of resources by welfare states challenges the structure of social stratification which in turn might drive authoritarians’ hostility toward the welfare component of the social market economy. Second, authoritarians tend to attribute the causes of poverty to a lack of individual effort rather than to structural causes (Bobbio et al., 2010). Consistent with Jost (2021) both arguments imply that, similar to SDO, there is a natural match between authoritarianism and acceptance of economic inequality (for a contrary position, see Malka & Soto, 2015). However, the results also highlight that the relationship between RWA, SDO, and support for capitalist market economies depends on the design of the system, as there are different varieties of capitalism that differ in the extent of market coordination and redistribution (Hall & Soskice, 2001).

### Limitations

Our findings are based on an observational study design. Therefore, conclusions about causal relationships between system-justifying ideologies and economic system support are not possible. Although we controlled for several important covariates, we cannot rule out the possibility that there are still unobserved factors that influence the relationship between RWA, SDO, and support for the social market economy. Only in the context of a randomized experimental design it can be ensured that the independent variables exert a causal effect on the dependent variables by manipulating one or more factors of interest and by controlling for confounding variables. Nevertheless, the findings of our study shed new light on the general validity of system justification theory, as the latter makes some predictions that cannot be confirmed by our results.

Furthermore, the attitude measure we used as a dependent variable does not correspond to the established ESJ scale. Our scale measures support for the German economic system in different facets, whereas the ESJ scale indicates the psychological defense of the economic system based on free-market principles. This difference in content could be a reason why we were not able to replicate the positive relationships between RWA, SDO and support for the free-market economy found in U.S. studies. However, we believe that the ESJ scale is not a pure measure of economic system support but rather incorporates elements of meritocratic beliefs that render dubious conclusions about associations with system-justifying attitudes.

## Conclusion

Our findings suggest that the relationship between support for the economic system of a country and system-justifying ideologies such as RWA and SDO is strongly dependent on the concrete configuration of the economic order, even if the economic system is based on market principles. Future research on system-justification theory should focus on cross-cultural designs that compare system-justifying tendencies in countries with liberal (e.g., USA) and social market economies (e.g., Austria, France, or Scandinavian countries) to test the robustness of our results. Generally, however, the results highlight the interplay of system-justifying ideologies with cultural, social, and economic conditions at the country level that should play a more prominent role in proving the cross-cultural validity of system-justification theory.

## Electronic supplementary material

Below is the link to the electronic supplementary material.

Supplementary Material 1

## Data Availability

The data that support the findings of this study is available from PsychArchives at 10.23668/psycharchives.12562.

## Footnotes

1 To ensure data quality, we also implemented two attention checks but with one exception, excluding inattentive participants does not substantially change the results. The association between RWA and diffuse support (see Table 1) decreases slightly in strength and no longer reaches conventional levels of statistical significance, *b* = 0.09, *SE* = 0.05. *t* = 1.90, *p* = .058, *N* = 800. However, this is due to the reduced number of cases and the associated loss of statistical power.

2 Because the three subscales are facets of the same construct, we estimated a path model in which we analyzed all three dependent variables simultaneously. As expected, both statistical approaches lead to similar results (for full results, see Supplementary Online Material, Table C-1).

3 The regression coefficients indicate the proportion of change in the dependent variable when the predictor variable moves from the lowest to the highest possible value. For example, an unstandardized regression coefficient of 0.20 means a 20% shift across the range of the dependent variable, when the predictor variable changes by one unit (from 0 to 1).
