# Supplementary material for: The association between system-justifying ideologies and attitudes toward the social market economy in Germany
Source: Curr Psychol. 2023 Mar 31:1–11. Online ahead of print. doi: 10.1007/s12144-023-04483-7 (PMC10064620; doi:10.1007/s12144-023-04483-7)
Supplement: Supplementary file 1 — Supplementary Material 1 [file 12144_2023_4483_MOESM1_ESM.pdf]

Supplemental Online Material

The Association between System-Justifying Ideologies and Attitudes toward the Social Market Economy in Germany

Alexander Jedinger & Simone Kaminski

https://doi.org/10.1007/s12144-023-04483-7

Table of Contents

Appendix A: Further Information about the Sample

Appendix B: Descriptive Statistics

Appendix C: Additional Path Analysis

Appendix A: Further Information about the Sample

Table A-1. Demographic Composition of the Sample Compared to the German Microcensus

	Sample (n = 886)	*German Microcensus (2019)*
**Gender**		
Male	53.2%	49.2%
Female	46.7%	50.8%
Diverse	0.1%	–
**Age**		
18-29	17.6%	18.9%
30-44	22.2%	22.2%
45-59	26.7%	26.8%
60+	33.4%	32.1%
**Education Level**		
Low (secondary qualification, after nine years of schooling)	33.9%	36.3%
Medium (intermediary secondary qualification, after ten years of schooling)	30.7%	30.0%
High (higher secondary qualification, after 12 or 13 years of schooling)	35.4%	33.5%
**Region**		
East Germany (incl. Berlin)	20.4%	20.8%
West Germany	79.6%	79.2%

Appendix B: Descriptive Statistics

Figure B-1. Violin Plot of Key Measures

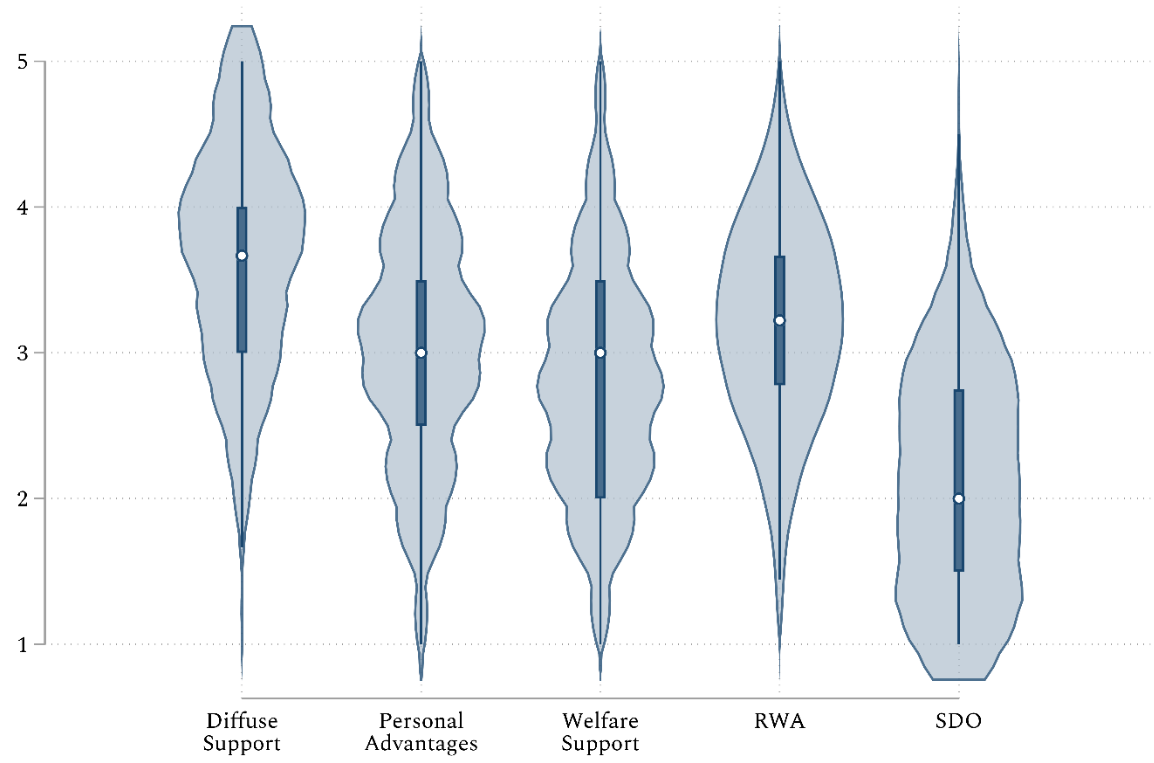

Appendix C: Additional Path Analysis

Table C-1. Path Model Predicting Attitudes toward the Social Market Economy

Predictor	Diffuse Support		Perceived Personal Advantages		Support for the Welfare Component	
	B (*SE*)	*Beta*	*B* (*SE*)	*Beta*	*B (SE)*	*Beta*
Right-wing Authoritarianism	0.11*	0.09	0.17***	0.13	-0.29***	-0.22
	(0.05)		(0.05)		(0.06)	
Social Dominance Orientation	-0.25***	-0.23	-0.16***	-0.14	-0.22***	-0.19
	(0.04)		(0.04)		(0.04)	
Individual Deprivation	-0.13***	-0.14	-0.18***	-0.18	-0.02	-0.02
	(0.04)		(0.04)		(0.04)	
Collective Deprivation	-0.14***	-0.14	-0.13**	-0.12	-0.08*	-0.08
	(0.04)		(0.04)		(0.04)	
Individual Relative Deprivation	-0.12**	-0.11	-0.33***	-0.28	-0.05	-0.04
	(0.04)		(0.04)		(0.04)	
Subjective Social Status	-0.01	-0.01	0.05	0.04	-0.02	-0.01
	(0.04)		(0.05)		(0.05)	
Household Income	0.03	0.04	0.00	0.01	-0.03	-0.04
	(0.02)		(0.02)		(0.02)	
Political Ideology	-0.03	-0.02	-0.10*	-0.07	-0.19***	-0.15
	(0.05)		(0.04)		(0.04)	
Political Interest	0.11***	0.13	0.04	0.04	0.04	0.04
	(0.03)		(0.03)		(0.03)	
Age	0.05	0.06	-0.01	-0.01	0.04	0.05
	(0.03)		(0.03)		(0.03)	
Male	0.04**	0.09	0.03	0.06	0.04**	0.09
	(0.01)		(0.01)		(0.02)	
Education (Ref.: Low Education)						
Medium Education	-0.04*	-0.09	-0.02	-0.05	0.01	0.01
	(0.02)		(0.02)		(0.02)	
High Education	0.01	0.03	0.02	0.05	0.04	0.08
	(0.02)		(0.02)		(0.02)	
East Germany	-0.04*	-0.08	-0.02	-0.03	-0.01	-0.01
	(0.02)		(0.02)		(0.02)	
Constant	0.79***		0.81***		0.80***	
	(0.06)		(0.05)		(0.06)	
R^2	0.26		0.32		0.23	

Note: The entries are unstandardized path coefficients (ML estimation), robust standard errors in parentheses, and standardized path coefficients. All continuous variables ranged from 0 to 1, $N = 886$. Standardized residual covariance of endogenous variables are .52, $p < .001$ (diffuse support and personal advantages), .07, $p = 0.13$ (diffuse support and welfare support), and .01, $p = 0.85$ (personal advantages and welfare support).
RWA = Right-wing authoritarianism, SDO = Social dominance orientation
* $p < .05$, ** $p < .01$, *** $p < .001$.
